# 

**Published:** 1904-03

**Authors:** 


### THE BRISTOL

### Medico-Chirurgical Journal.

A JOURNAL OF THE MEDICAL SCIENCES FOR THE
WEST OF ENGLAND AND SOUTH WALES

*PUBLISHED UNDER THE AUSPICES OF*

*THE BRISTOL MEDICO-CHIRURGICAL SOCIETY.*

EDITOR:
R. SHINGLETON SMITH, M.D., B.Sc.,

WITH WHOM ARE ASSOCIATED
J. MICHELL CLARKE, M.A., M.D., J. LACY FIRTH, M.S., M.D.,
AND JAMES SWAIN, M.S., M.D.

ASSISTANT-EDITOR:
P. WATSON WILLIAMS, M.D.

EDITORIAL SECRETARY:
JAMES TAYLOR.

---

"Scire est nescire, nisi id me
Scire alius sciret."

---

VOL. XXII.

LIBRARY

SURGEON GENERAL'S OFFICE

MAR.-1--1906

190392.

BRISTOL: J. W. ARROWSMITH.
LONDON: J. & A. CHURCHILL, 7 GREAT MARLBOROUGH STREET.
1904.

WI
BR323

J.W. Arrowsmith

Publisher

Bristol.

33754

### CONTENTS OF VOLUME XXII.

Page

Leprosy in Jamaica. By W. D. Neish, M.D., and T. J. Tonkin, L.R.C.P., L.R.C.S. Edin. ... ... ... ... ... ... 1

Subungual Exostosis. By W. Roger Williams, F.R.C.S. ... ... 17

Aneurysm of the Ascending Aorta. By F. H. Edgeworth, M.B. Cantab., D.Sc. Lond. ... ... ... ... ... ... 23

The Vagaries of Abdominal Tuberculosis: A Clinical Study. By James Swain, M.S., M.D. Lond., F.R.C.S. ... ... ... 31

The Present Position of Radiotherapy in Therapeutics. By W. Kenneth Wills, M.B., B.C. Cantab. ... ... ... ... 39

A Case of Tubal Gestation Producing Severe Hemorrhage without Rupture, Associated with the Presence of an Accessory Fallopian Tube. By Ernest W. Hey Groves, M.D., B.Sc. Lond. .. 46

Ethyl Chloride as a General Anæsthetic. By C. Hamilton Whiteford, M.R.C.S., L.R.C.P. ... ... ... ... ... ... 49

Note on the Subsequent History of a Case of Pylorectomy. By A. W. Prichard ... ... ... ... ... ... ... 53

Mortality and Sickness in the City of Bristol for the Year 1903. By D. S. Davies, M.D. Lond. ... ... ... ... ... ... 55

Forms of Joint Disease met with in Medical Practice. By Wm. Hale White, M.D. Lond., F.R.C.P. ... ... ... ... 97

Notes on some Cases from an Electrical Department. By George Parker, M.A., M.D. Camb. ... ... ... ... ... ... 112

A Case of Cæsarean Section for Obstruction of Labour by a Pelvic Tumour. By W. C. Swayne, M.D., B.S. Lond. ... ... ... 120

A Case of Congenital Hypertrophic Stenosis of the Pylorus. By Theodore Fisher, M.D. Lond., M.R.C.P., and Newman Neild, M.B., Ch.B. Vict. ... ... ... ... ... ... ... 123

A Case of Æstivo-Autumnal Malarial Fever with Parasites of an Unusual Type. By J. O. Symes, M.D. Lond., D.P.H. ... 126

Notes on a Case of Blackwater Fever. By E. Cecil Williams, B.A., M.B. Cantab. ... ... ... ... ... ... ... 128

Lac Vinum Infantum: A Review of the Work of Infant Milk Depôts. By J. M. Fortescue-Brickdale, M.A., M.D. Oxon. ... ... 196

CONTENTS.

Page

Some Observations on Tuberculous Disease of the Hip-Joint in Childhood and Youth. By CHARLES A. MORTON, F.R.C.S. ... 207

On the Throat as the Source of Systemic Infection in Acute Rheumatism. By P. WATSON WILLIAMS, M.D. Lond. ... ... 215

The Indications for Operation in Myo-Fibroma of the Uterus. By JAMES SWAIN, M.S., M.D. Lond., F.R.C.S. ... ... ... 220

Ethyl-Chloride: A Few Practical Remarks. By A. L. FLEMMING 228

The Induction of General Anæsthesia by Intra-Spinal Injections of Cocaine. By ALFRED S. GUBB, M.D. Paris, M.R.C.S., &c. ... 231

On Surgical Analgesia by Spinal Cocainisation. By WILLIAM JONES GREER, F.R.C.S.I., D.P.H.I. ... ... ... ... 235

The Relationship of Chorea and Rheumatism. By JOS. J. S. LUCAS, M.B., B.A. Lond. ... ... ... ... ... ... ... 239

Hæmatemesis Associated with Small White Kidneys. By THEODORE FISHER, M.D., M.R.C.P. ... ... ... ... ... ... 243

Notes on a Case of Infantilism. By E. CECIL WILLIAMS, B.A., M.B. Cantab. ... ... ... ... ... ... ... ... 247

Problems in Psychology. By REGINALD EAGER, M.D. Lond. ... 289

The Long Fox Lecture. By JOHN BEDDOE, M.D., LL.D., F.R.S.... 303

On Torsion of the Spermatic Cord, with the Report of a Recent Case. By J. LACY FIRTH, M.S. Lond., F.R.C.S. ... ... 320

Case of Mesenteric Cyst. By A. W. PRICHARD ... ... ... 332

PROGRESS OF THE MEDICAL SCIENCES ... ... 58, 131, 249, 334

REVIEWS OF BOOKS ... ... ... ... ... 68, 145, 257, 347

EDITORIAL NOTES ... ... ... ... ... 74, 175, 268, 362

NOTES ON PREPARATIONS FOR THE SICK ... ... 81, 181, 276, 366

THE LIBRARY OF THE BRISTOL MEDICO-CHIRURGICAL SOCIETY ... ... ... ... 85, 185, 283, 374

MEETINGS OF SOCIETIES ... ... ... ... ... 87, 188, 377

LOCAL MEDICAL NOTES ... ... ... ... 94, 191, 288, 382

OBITUARY ... ... ... ... ... ... ... ... 193, 286

acceptable and well borne in gastro-enteritis. A pint of the solution drunk daily seemed to have excellent results as an internal antiseptic, but we cannot speak from personal experience of its use in typhoid fever, for which it is recommended administered in this manner.

**Terpin Hydrate and Heroin Co.** in chocolate-coated tablets is an agreeable form in which to prescribe this useful combination.

### The Library of the Bristol Medico-Chirurgical Society.

*The following donations have been received since the publication of the List in December :*

*February 29th, 1904.*

| | |
|---|---|
| The Director of the Department of Public Health and Charities, Philadelphia (1)... ... ... ... | 1 volume. |
| L. M. Griffiths (2) ... ... ... ... ... ... ... | 2 volumes. |
| Medical and Chirurgical Faculty of the State of Maryland (3) ... ... ... ... ... ... ... | 1 volume. |
| Medical Library of McGill University (4) ... ... | 2 volumes. |
| The Medical Officer of the London County Council (5) ... ... ... ... ... ... ... ... | 3 ,, |
| New York Academy of Medicine (6) ... ... ... | 1 volume. |
| The Council of the Otological Society (7) ... ... | 1 ,, |
| The Council of the Pathological Society of London (8) | 1 ,, |
| The Scholastic Trading Company (9) ... ... ... | 1 ,, |
| R. Shingleton Smith, M.D. (10) ... ... ... ... | 1 ,, |
| James Swain, M.D. (11) ... ... ... ... ... ... | 1 ,, |

Pamphlets have been received from Mr. L. M. Griffiths and the Medical Library of McGill University. Unbound periodicals have been presented by Mr. J. Paul Bush.

### FIFTY-FIRST LIST OF BOOKS.

The titles of books mentioned in previous lists are not repeated.

The figures in brackets refer to the figures after the names of the donors, and show by whom the volumes were presented. The books to which no such figures are attached have either been bought from the Library Fund or received through the *Journal*.

*Atlas of Illustrations of Clinical Medicine, Surgery and Pathology, An.* Fasc. VIII., IX.... ... ... ... ... ... ... 1903
**Bastian, H. C.** ... *Studies in Heterogenesis* ... ... ... ... ... ... .. 1903
**Beckmann, H.** ... *Das Eindringen der Tuberculose* ... ... ... ... ... ... 1904
**Eenson, J. R.** ... *A Scheme for the Provision of Nursing Homes for the Middle Classes* ... ... ... ... ... ... ... 1904
**Bowles, R. L.** ... *The Treatment of the Apparently Drowned* ... ... ... 1904
**Collie, A.** ... ... *The Infectivity of Enteric Fever* ... ... ... ... ... ... 1904
**Cunning, J.** ... *Aids to Surgery* ... ... ... ... ... ... ... ... ... 1904
*Eastern Hospitals and English Nurses.* 2 vols. ... ... ... (2) 2nd Ed. 1856
**Fenwick, E. H.** ... *Ureteric Meatoscopy in Obscure Diseases of the Kidney* 1903
**Francis, A.** ... ... *Asthma in relation to the Nose* ... ... .. ... ... 1903
**Frank, F.** ... ... *Geburtshilfliche Polikliniken an Hebammenschulen* ... 1904
**Hare, F.** ... ... *Mechanism of the Paroxysmal Neuroses* ... ... ... 1903
**Herring, H. T.** ... *The Sterilisation of Urethral Instruments* ... ... ... 1903
**Hillier, A.** ... ... *The Prevention of Consumption.* (Revised by R. Koch) 1903
**Homfray, C. T. Kingzett and D.** *A Pocket Dictionary of Hygiene* 2nd Ed. 1904
**Kingzett and D. Homfray, C. T.** *A Pocket Dictionary of Hygiene.* 2nd Ed. 1904
**Langenhagen, M. de.** *Muco-membranous Entero-colitis* ... ... ... (10) 1903
**Melland, C. H.** ... *Clinical Methods* ... ... ... ... ... ... ... ... ... 1903
**Miles, A. Thomson and A.** *Manual of Surgery* Vol. I. ... ... ... ... 1904
**Milroy, J. A. and T. H.** *Practical Physiological Chemistry* ... ... ... ... 1904
**Overall, G. W.** ... *A Non-Surgical Treatise on Diseases of the Prostate Gland and Adnexa* ... ... ... ... ... ... [1903]
**Reinhardt and D. Thomas, C.** *A Handbook of the Open-Air Treatment* 2nd Ed. 1902
**Robinson, M.** ... *A Guide to Urine Testing* ... ... ... ... 2nd Ed. 1904
**Robson, A. W. M.** *Diseases of the Gall-Bladder and Bile-Ducts* 3rd Ed. 1904
**Shaw, J.** ... ... *Mental Diseases and Degeneracy* ... ... ... ... ... 1903
**Smith, E. N.** ... *Lateral Curvature, Stooping, &c.* ... ... ... ... ... 1904
**Stacpoole, Florence** *Ailments of Women and Girls* ... ... ... .. ... 1904
**Sutherland, W. G.** *Dispensing made Easy* ... ... ... ... ... ... ... 1903
**Thomas, C. Reinhardt and D.** *A Handbook of the Open-Air Treatment* 2nd Ed. 1902
**Thomson and A. Miles, A.** *A Manual of Surgery* Vol. I. ... ... ... 1904
**Treves, Sir F.** ... *A Manual of Operative Surgery.* 2 vols. New [2nd] Ed. 1903
**Walker, E. W. A.** *The General Pathology of Inflammation, Infection and Fever* ... ... ... ... ... ... ... ... .. ... 1904
**Waring, H. J.** ... *Manual of Operative Surgery* ... ... ... ... 2nd Ed. 1904
**Williams, W. R.** *Vaginal Tumours* ... ... ... ... ... ... ... ... 1904
**Willoughby, E. F.** *Milk : its Production and Uses* ... ... ... ... ... 1903

### TRANSACTIONS, REPORTS, JOURNALS, &c.

American Dermatological Association, Transactions of the ... ... ... 1903
Association of American Physicians, Transactions of the Vol. XVIII. 1903
British Almanac, The ... ... ... ... ... ... ... ... ... ... ... ... 1904
British Medical Journal, The ... ... ... ... ... ... ... ... Vol. II. for 1903
Clinical Journal, The ... ... ... ... ... ... ... ... ... Vol. XXII. 1903
Edinburgh Medical Journal, The ... ... ... ... ... ... N.S., Vol. XIV. 1903
Hazell's Annual ... ... ... ... ... ... ... ... ... ... ... ... ... .. ... 1904

Hospitals—
Medical and Surgical Reports of the Boston City Hospital (11) 14th series 1903
Johns Hopkins Hospital Reports, The ... ... ... ... Vol. XI. 1903
King's College Hospital Reports ... ... Vols. VI.—VIII. 1900-03
Philadelphia Hospital Reports ... ... ... ... ... (1) Vol. V. 1903
Studies from the Royal Victoria Hospital, Montreal. (4) Vol. I., Nos. 4, 5 1902-03
Saint Bartholomew's Hospital Reports ... ... ...Vol. XXXIX. 1904
Journal of Mental Pathology, The... ... ... ... ...Vols. III., IV. 1902-03
Lancet, The ... ... ... ... ... ... ... ... ... ... Vol. II. for 1903
Library World, The ... ... ... ... ... ... ... ... ... ... Vol. V. 1903
London County Council, Annual Reports of the Medical Officer of Health of the, 1900-02 .. ... ... ... ... ... ... ... (5) 1900-03
Medical and Chirurgical Faculty of the State of Maryland, Transactions of the .. .. ... ... ... ... ... ... ... ... .. ... ... (3) 1903
Medical Directory, The ... ... .. ... ... ... ... ... ... ... ... 1904
Medical Society's Transactions, The ... ... ... ... ... Vol. XXVI. 1903
New Hampshire Medical Society, Transactions of the ... ... ... ... 1903
New York Academy of Medicine, Transactions of the, 1896—1901. (6) 1903
Ophthalmological Transactions ... ... ... Vols. XXII., XXIII. 1902-03
Otological Society of the United Kingdom, Transactions of the (7) Vol. IV. 1903
Pathological Society of London, Transactions of the ...(8) Vol. LIV. 1903
Philadelphia Medical Journal, The ... ... ... ... ... ... Vol. XI. 1903
Public Health ... ... ... ... ... ... ... ... ... ... Vol. XV. 1902-03
Publishers' Circular, The... ... ... ... ... ... (9) Vol. LXXVIII. 1903
Royal Academy of Medicine in Ireland, Transactions of the Vol. XXI. 1903
Sanitary Record, The ... ... ... ... ... ... ... ... Vol. XXXI. 1903
Smithsonian Institution, Annual Report of the, 1902... ... ... ... ... 1903
Whitaker's Almanac ... ... ... ... ... ... ... ... ... ... ... ... 1904
Who 's Who ... ... ... ... ... ... ... ... ... . . ... ... ... ... 1904

---

### MEETINGS OF SOCIETIES.

### Bristol Medico-Chirurgical Society.

### Local Medical Notes.

The following rules for the prevention of **Consumption or Tuberculosis** have been issued by direction of the Health Committee of the City and County of Bristol:—

Any person may catch Consumption from another, especially if living in the same room or house with a consumptive patient; *but with care there need be no danger.*

**The spit, phlegm, or expectoration of a consumptive patient is full of poisonous germs** which are the cause of Consumption; this may re-infect the patient himself, therefore these precautions will promote his own recovery.

The patient must take unceasing care never to allow his spit to be deposited upon his handkerchief, clothing or bedding, or upon anything, such as the floor or walls, where it may afterwards dry up and become scattered about as dust. People breathing in or swallowing this dust are in danger of catching Consumption.

Patients suffering from Consumption must always spit into a small spittoon or cup containing some water or disinfecting fluid, or into pieces of rag or paper, which should be burnt before the spit on them has become dry.

The contents of the spittoon or cup should be poured down the water closet, or into the back of the kitchen fire, after which the spittoon or cup should be disinfected by boiling it in water.

No one must spit on the floor inside any building or in any public conveyance; spitting in the street should be avoided.

If any **handkerchiefs, clothing**, etc., have become soiled with the patient's spit, they **must be carefully disinfected** by boiling in water or by soaking for half an hour in some of the disinfecting fluid.

A consumptive patient should not kiss anyone on the mouth.

A consumptive mother should not suckle her baby.

**Nothing which has touched the mouth of a consumptive patient, such as a spoon, fork, cup, pipe, etc., should be used by another person until it has been thoroughly washed with boiling water.**

Persons attending a consumptive patient should be very careful to wash and scrub their hands thoroughly after touching the patient, or anything connected with the patient which may have been soiled by the spit.

The bowel discharges of a consumptive patient should be received into a disinfectant, and the utensil thoroughly scalded on each occasion after it has been emptied.

### THE BEDROOM OF A CONSUMPTIVE PATIENT.

No other person should sleep in the same room, but persons may safely inhabit the same house if reasonable precautions are observed.

**The dusting and cleansing of the room** must be carried out frequently and with the greatest care. The dust is dangerous, since it may contain disease germs. **Wet dusters** must be used to wipe up the dust on the floor, furniture, woodwork, etc., and must afterwards be boiled. Tea leaves used on the floor should afterwards be burnt. Cleansing should be done frequently.

**Admit as much sunshine and fresh air as possible** into the room. These are the best remedies for curing, as well as preventing, Consumption.

No one should occupy the bedroom or any room which has been constantly used by a consumptive patient until it has been thoroughly disinfected and cleansed. This disinfection will be carried out by the Medical Officer of Health, on written application to the Offices, 40 Prince Street, Bristol.

---

**Conditions which make persons liable to Consumption.**

1. The commonest condition is probably the stuffy, poisonous air of unventilated bedrooms, living-rooms, and work-rooms. Therefore **rooms should never be overcrowded, chimneys never blocked up. So far as possible windows should always be kept widely opened day and night throughout the year.** Prevent cross draughts by screens, etc., and provide warmer clothing and bedding for the inmates if they feel the cold.
2. Prolonged attacks of cough and cold. In such cases do not fail to consult a doctor.
3. Absence of sunlight and fresh air.
4. Everything which lowers the general health—intemperance, damp dwellings, unventilated rooms.

**Milk** may carry the poison of Consumption ; therefore all milk should be boiled or scalded. Meat should be properly cooked.

**Bye-law adopted by the Bristol City Council on November 24th, 1903 :—**

"A person shall not spit on the floor, side, or wall of any public carriage, or of any public hall, public waitingroom, or place of public entertainment, whether admission thereto be obtained upon payment or not Any person offending against this Bye-law shall be liable to a fine not exceeding £1."

D. S. DAVIES, M.D.,

*December, 1903.* *Medical Officer of Health.*

**University College, Bristol.**—In the recent M.B. Examination for Honours in the University of London, Mr. A. R. Short obtained the first place in Medicine, with the Gold Medal and Scholarship, and also First Class Honours in Obstetric Medicine, Mr. J. J. S. Lucas obtained Third-Class Honours in Medicine and Forensic Medicine. In the final B.S. Examination of the same University, Mr. M. H. Phillips gained the Gold Medal and Scholarship in Surgery. In the final F.R.C.S. England Examination Mr. M. H. Phillips was successful.

**The Royal National Pension Fund for Nurses,** which was inaugurated in 1887, was established under the Presidency of Her Royal Highness the Princess of Wales, with his Royal Highness the Prince of Wales as Patron. On his accession, His Majesty King Edward VII. was graciously pleased to continue his patronage of the Fund, and Her Majesty Queen Alexandra graciously accepted the office of President. The chief object of the Fund is to afford to Nurses an absolutely safe means of providing, at the lowest possible cost to themselves, an allowance during incapacity for work caused by sickness or accident, and a certain income for their declining years. This object will be carried out by receiving and investing such fixed periodical sums as those who join the Fund can afford, and by adding to the pensions all the profits arising from any source, and by supplementing these sums from a Donation Bonus Fund, created and maintained by those interested in Nurses and Nursing Institutions. In order to encourage the "Sisters" and Members of the Private Nursing Staff of the Bristol Royal Infirmary to make some provision for a certain income in the future, the Committee of the Infirmary have joined the Royal National Pension Fund for Nurses, and are prepared to assist any Sister or Nurse who may wish to join the Fund to the extent stated in and according to the Regulations. The Committee feel that in taking this step they are providing for the Sisters and Nurses a safe investment to secure the intended result. The Fund is practically a Mutual Life Assurance Society, all the profits going to the Nurses. By joining on the "Returnable Scale," Nurses may have their premiums returned to them with compound interest, or if they prefer to pay for a pension they receive substantial additions from the profits arising from the working of the Fund and also from the Donation Bonus Fund.

